# Clinical analysis of redo aortic root replacement after cardiac surgery: a retrospective study

**DOI:** 10.1186/s13019-021-01587-8

**Published:** 2021-07-28

**Authors:** Jianying Deng, Qianjin Zhong

**Affiliations:** 1 Department of Cardiovascular Surgery, Chongqing Kanghua Zhonglian Cardiovascular Hospital, 168 Haier Street, Jiangbei District, Chongqing, 400015 China; 2 Department of Cardiovascular Surgery, Army Medical Center of PLA, Chongqing, 400020 China

**Keywords:** Aortic root replacement, Redo cardiac procedure, Aortic disease, Surgery

## Abstract

**Objectives:**

To explore the etiology, previous cardiac procedure methods and outcomes of redo aortic root replacement after cardiac surgery.

**Methods:**

A retrospective analysis of 41 patients who underwent aortic root replacement surgery in our hospital from February 2010 to February 2020 who underwent at least one cardiac surgery in the past, including 27 males and 14 females, with an average age of 49.5 ± 10.2 years old. Indications for reoperation include: aortic sinus dilation and ascending aortic aneurysm in 20 cases (48.8%), recurrent aortic dissection in 7 cases (17.1%), pseudoaneurysm of aortic root in 4 cases (9.8%), prosthetic valve endocarditis in 5 cases (12.2%) and paravalvular leakage in 5 cases (12.2%). According to whether the previous procedure involved aortic root surgery, they were divided into 2 groups, namely aortic root surgery-involved (ARS) group and non-aortic root surgery-involved (NRS) group. After the patients were discharged from hospitals, follow-ups were carried out through outpatient clinic or telephone for 5 years. Kaplan-Meier was used for survival analysis.

**Results:**

All patients underwent Bentall procedure with a median sternum incision. Six patients (14.6%) died during the postoperative hospitalization and 3 patients (8.6%) died during the follow-up. The 1-year, 3-year, and 5-year survival in ARS group were 92.6, 92.6, and 92.6%, respectively; the 1-year, 3-year, and 5-year survival in NRS group were 100, 85.7, and 85.7%, respectively. There was no statistical difference between the two groups in the cause of redo aortic root replacement, procedure time, postoperative complications, postoperative hospital stay, hospital mortality, and 5-year cumulative survival (*p* > 0.05).

**Conclusions:**

Redo aortic root replacement is difficult and high risk. Bentall procedure is still a reliable surgical option for redo aortic root replacement, with good short- and mid-term results. The prognosis of redo aortic root replacement is not necessarily related to the etiology of patient’s surgery and the methods of previous cardiac procedure.

## Background

In 1968, Bentall and De Bono first reported the use of a Teflon graft and Starr valve for aortic root replacement to treat aortic root diseases [1]. Since then, the success rate of aortic root surgery has been significantly improved, and a new era of aortic surgery has been opened. Subsequently, Cabrol and Kouchoukos each improved the technical details of the classic Bentall surgery. At present, Bentall procedure has become the standard procedure for the treatment of aortic root aneurysms and aortic A-type dissection with aortic regurgitation. The advantages of Bentall procedure are that the postoperative effect is good, the diseased aortic tissue can be completely removed, the operation is relatively simple, and it is easy to promote. The disadvantages are that it may require lifelong anticoagulation after procedure, and the coronary artery opening may form a true or false aneurysm.

In some patients who needed redo caidiac surgery with normal aortic roots, there is no need to deal with aortic roots. However, in some patients with abnormal aortic roots, such as aortic root aneurysm, aortic root abscess, severe calcification of the aortic root and dilated aortic annulus, Bentall procedure is a safe and reliable option. This article summarizes the preoperative, postoperative and follow-up results of patients undergoing redo Bentall procedure after cardiac surgery in our hospitals.

## Methods

### Clinical information

From February 2010 to February 2020, 41 patients in our hospitals underwent redo Bentall procedure, including 27 males and 14 females, aged 16–72 (49.5 ± 10.2) years old. In these cases, at least one cardiac procedure has been performed, and 6 of them had a history of two cardiac procedures performed in the past. The interval between the previous cardiac operation and this cardiac surgery is 8 months to 25 years, with an average of (7.0 ± 7.5) years. Ten patients had a history of hypertension. The preoperative electrocardiogram (ECG) showed atrial fibrillation in 6 patients. Four patients underwent emergency surgery, 2 patients had prosthetic valve endocarditis (PVE) after aortic valve replacement, which caused acute left heart failure, the other 2 patients had acute aortic dissection after aortic valve replacement.

### Surgical approach

**Fig. 1:**
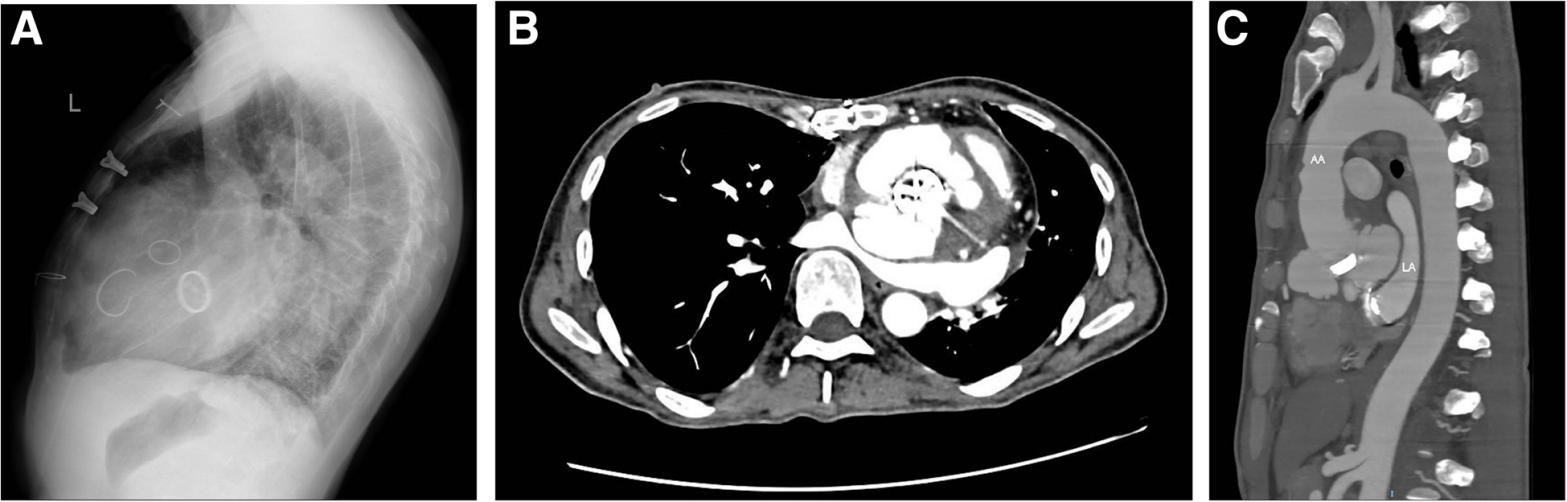
Lateral chest X-ray (**A**) indicates that the right ventricle is closely attached to the sternum, axial CT image of the chest (**B**) indicates that the distance between the aortic root pseudoaneurysm and the sternum is small, and sagittal CT image (**C**) shows the ascending aorta is closely attached to the sternum

**Fig. 2:**
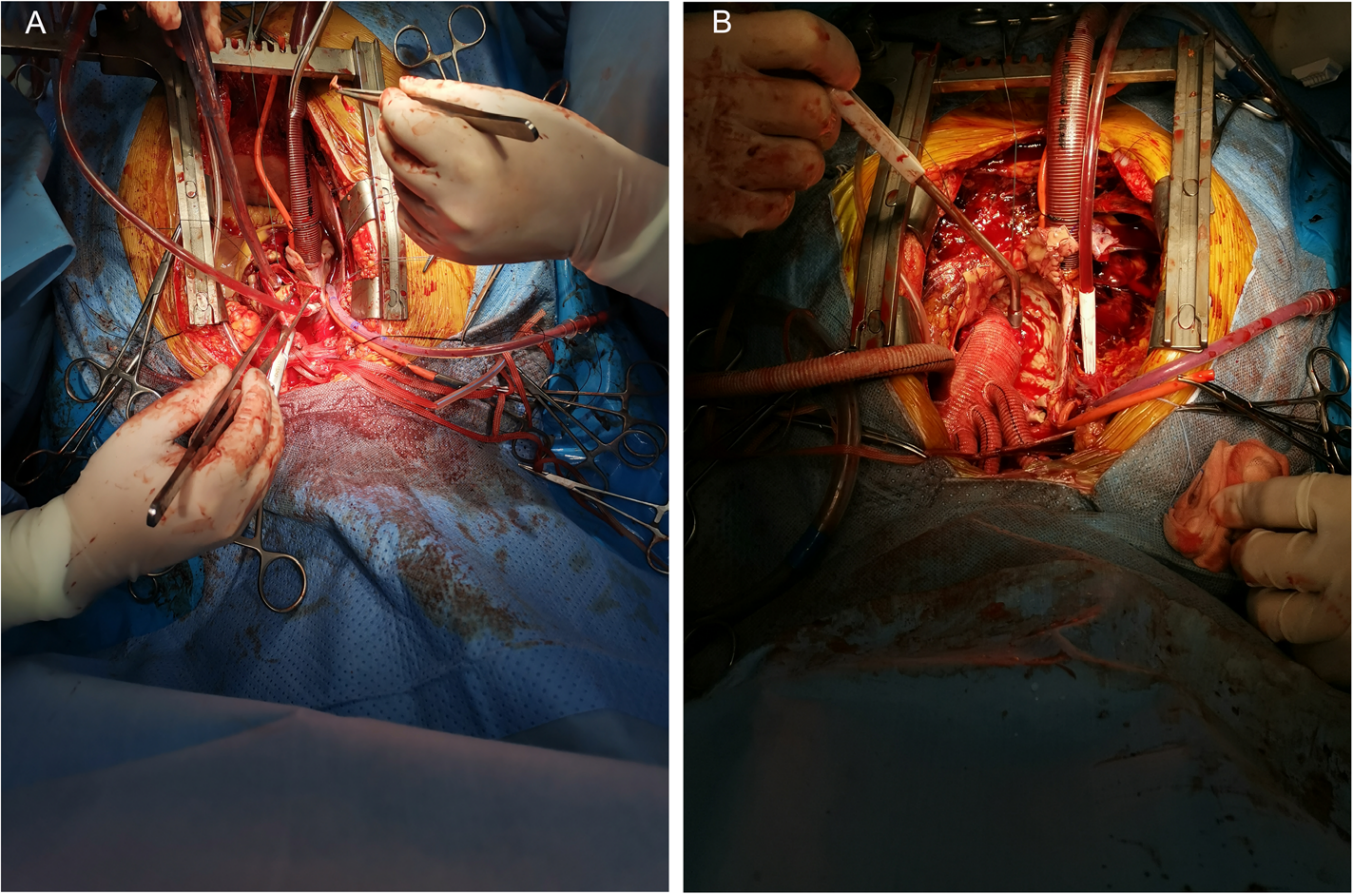
Aortic dissection involves the right coronary sinus, and the right coronary artery opening is avulsed (**A**); some redo aortic root replacement patients underwent Sun’s surgery simutaneously (**B**)

All forty-one patients underwent thoracotomy with a median sternum incision. During the establishment of cardiopulmonary bypass (CPB), only 2 cases were intubated through the ascending aorta, and the remaining 39 cases were intubated through the femoral artery. After thoracotomy, 4 patients underwent axillary artery or innominate artery cannulation according to their condition. The femoral vein or superior and inferior vena cava cannulation was selected according to the specific conditions of these patients, and CPB was established by right atrium cannulation in 4 patients. The most important issue for reoperation is to safely perform thoracotomy to avoid death due to hemorrhage. Our principle is to evaluate the distance between the sternum and the aorta and right ventricle through imaging examinations before surgery (Fig. 1). When the distance is close or the right ventricular pressure is high, peripheral CPB is established first to avoid heart injury and hemorrhage during thoracotomy. Fortunately, none of these patients experienced severe bleeding or other complications when sawing the sternum. In terms of myocardial protection, except for one patient who received intermittent retrograde perfusion with cold-blooded myocardial protective solution due to pulsating pseudoaneurysm of the aortic root, the rest were all intermittent antegrade perfusion with medium-low temperature cardiopulmonary bypass, and the lowest body temperature was 24.7 ~ 32.8 (30.7 ± 1.9)°C. All patients underwent Bentall procedure, and to avoid reoperation, artificial mechanical valves were used. For coronary artery transplantation, if the adhesion around the coronary artery opening is severe and it is difficult to dissociate, and the coronary artery opening is not obviously displaced, we used the direct anastomosis method in 4 cases (13%), and the rest of 37 cases (87%) were carefully dissected to isolate the coronary artery opening and use button-shaped anastomosis method. One patient underwent a great saphenous vein anastomosis to lengthen the right coronary artery due to difficulty in right coronary artery anastomosis, and one underwent a bypass from the great saphenous vein to the proximal right coronary artery due to the narrow opening of the right coronary artery, and the right coronary artery opening was closed at the same time. During the same period, 3 cases of mitral valve replacement, 4 cases of mitral valvuloplasty, 8 cases of tricuspid valvuloplasty, 4 cases of coronary artery bypass grafting, and 3 cases of Sun’s operation (Fig. 2).

### Postoperative follow-up methods

Follow-ups were conducted at 1 month, 3 months, 6 months, 12 months, 24 months, 36 months, 48 months, and 60 months after discharge through outpatient clinics and/or telephone calls. Follow-up content includes patient information, survival status, clinical symptoms and prognostic factors. The 5-year survival was defined as the proportion of patients who survived 5 years or more from the date of discharge. Lost to follow-up is defined as not seeing a doctor at outpatient clinic or not answering calls during the follow-up period, and not answering calls for 3 consecutive days.

### Statistical analysis

The SPSS 22.0 statistical software was used to analyze the data. The quantitative data of the normal distribution is expressed as ^−^x ± s, and the independent sample *t* test is used for the comparison between groups. Count data is expressed in frequency and percentage, and comparisons between groups are performed by χ^2^ test or Fisher’s exact probability method. Kaplan-Meier curve was used for survival analysis. *p* < 0.05 was considered statistically significant.

## Results

### Patient characteristics

The previous cardiac surgery of 41 patients was as follows: 4 cases of Bentall (including 3 cases of Sun’s procedure at the same time), 21 cases of aortic valve replacement (including 2 cases of biological valves, 15 cases of mitral valve replacement or mitral valvuloplasty or tricuspid valvuloplasty at the same time, 2 cases of ascending aorta angioplasty, and 2 cases of aortic root widening), 4 cases of aortic valvuloplasty (including 2 cases of type A preexcitation syndrome abnormal bypass conduction amputation + ventricular septal defect repair + patent foramen ovale repair + mitral valvuloplasty), 2 cases of aortic root replacement with aortic valve-preserving, 2 cases of Sun’s procedure, 3 cases of mitral valve replacement, 3 cases of radical operation for Tetralogy of Fallot, and 2 cases of ventricular septal defect repair.

According to whether the previous procedure involved aortic root surgery, they were divided into 2 groups, namely aortic root surgery-involved (ARS) group and non-aortic root surgery-involved (NRS) group. Based on the above grouping, there were 31 patients in ARS group, and 10 patients were enrolled in NRS group. There was no statistical difference in preoperative characteristics between the two groups (Table 1).

**Table 1:** Preoperative characteristics of 41 patients [cases(%)/^−^x ± s]

Clinical data	Full sample(*n* = 41)	ARS(*n* = 31)	NRS(*n* = 10)	*P* value
Age	49.5 ± 10.2	49.5 ± 10.1	49.8 ± 10.5	0.838
Gender				0.750
Male	27 (65.9)	20 (64.5)	7 (70)	
Female	14 (34.1)	11 (35.5)	3 (30)	
BMI(kg/m^2^)	21.5 ± 4.2	21.7 ± 4.1	21.4 ± 4.3	0.919
Hypertension	10 (24.4)	7 (22.6)	3 (30)	0.635
Diabetes	4 (9.8)	3 (9.7)	1 (10.0)	0.976
Hyperlipidemia	5 (12.2)	4 (12.9)	1 (10.0)	0.807
COPD	2 (4.9)	2 (6.5)	0 (0)	0.410
History of stroke	2 (4.9)	2 (6.5)	0 (0)	0.410
Preoperative hemodialysis	1 (2.4)	1 (3.2)	0 (0)	0.565
Peripheral vascular disease	4 (9.8)	3 (9.7)	1 (10)	0.976
Coronary heart disease	4 (9.8)	3 (9.7)	1 (10)	0.976
Atrial fibrillation	6 (14.6)	5 (16.1)	1 (10)	0.633
Marfan syndrome	2 (4.9)	2 (6.5)	0 (0)	0.410
Recurrent aortic dissection	7 (17.1)	4 (12.9)	3 (30)	0.270
Heart function classification (NYHA)				0.953
I/II	12 (29.3)	9 (29.0)	3 (30)	
III/IV	29 (70.7)	22 (71.0)	7 (70)	
Left ventricular ejection fraction < 50%	7 (17.1)	5 (16.1)	2 (20)	0.777
Emergency surgery	4 (9.8)	3 (9.7)	1 (10)	0.976
Previous cardiac surgery				0.000
Bentall procedure	4 (9.8)	4 (12.9)	0 (0)	
Aortic valve replacement	21 (51.2)	21 (67.7)	0 (0)	
Aortic valvuloplasty	4 (9.8)	4 (12.9)	0 (0)	
Aortic root replacement	2 (4.9)	2 (6.5)	0 (0)	
Sun’s procedure	2 (4.9)	0 (0)	2 (20)	
Mitral valve replacement	3 (7.3)	0 (0)	3 (30)	
Tetralogy of Fallot repair	3 (7.3)	0 (0)	3 (30)	
Ventricular septal defect repair	2 (4.9)	0 (0)	2 (20)	

Abbreviations: *BMI* Body Mass Index; *COPD* chronic obstructive pulmonary disease; ARS,aortic root surgery-involved; *NRS* non-aortic root surgery-involved

Note: Values are presented as n (%) or mean ± standard deviation

Forty-one patients underwent redo aortic root replacement for the following etiologies: aortic root dilation and ascending aortic aneurysm in 20 cases, recurrent aortic dissection in 7 cases (3 cases of Debakey Iand 4 cases of Debakey II), 4 cases of aortic root pseudoaneurysm (all after Bentall procedure, 2 cases had coronary anastomotic tear, 2 cases had valved catheter prosthesis and aortic root tear), 5 cases of PVE (3 cases of aortic root abscess and 2 cases of severe calcification of the aortic sinus wall), and 5 cases of paravalvular leakage (PVL) (4 cases of difficulty in suture of aortic annulus due to Behcet’s disease, 1 case of aortic sinus wall injury due to failure of interventional closure of paravalvular leak). There was no statistical difference in the etiologies of redo aortic root replacement between the two groups (Table 2).

**Table 2:** Etiologies of redo aortic root replacement[cases(%)]

Variables	Full sample(*n* = 41)	ARS(*n* = 31)	NRS(*n* = 10)	*P* value
Aortic sinus dilation/Ascending aortic aneurysm	20 (48.8)	13 (41.9)	7 (70)	0.123
Recurrent aortic dissection	7 (17.1)	4 (12.9)	3 (30)	0.270
Debakey I	3 (7.3)	1 (3.2)	2 (20)	
Debakey II	4 (9.8)	3 (9.7)	1 (10)	
Aortic root pseudoaneurysm	4 (9.8)	4 (12.9)	0 (0)	0.232
Prosthetic valve endocarditis	5 (12.2)	5 (16.1)	0 (0)	0.175
Paravalvular leakage	5 (12.2)	5 (16.1)	0 (0)	0.175

Note: Values are presented as n (%)

### Perioperative results

The hospital mortality is 14.6% (6/41). The main causes of death included 3 deaths from postoperative heart failure and 3 deaths from septic shock (including 2 cases of mediastinal infection and 1 case of pulmonary infection). Of the 6 deaths, 4 ceses (12.9%) were in the ARS group (3 cases of septic shock and 1 case of heart failure), and 2 cases (20%) were in the NRS group (2 cases of heart failure). There was no statistical difference in the hospital mortality between the two groups (*p* = 0.581).

The CPB time in the ARS group was (169.3 ± 42.1) min and the aortic block time was (85.6 ± 22.8) min, while the CPB time in the NRS group was (168.0 ± 41.1) min, and the aortic block time was (85.4 ± 22.2))min. There are no statistical differences between the two groups (*p >* 0.05).

Four patients (9.8%) underwent a re-exploratory thoracotomy due to a large amount of pleural fluid in the early postoperative period, including 3 cases in the ARS group and 1 case in the NRS group. There were 2 cases (4.9%) of stroke, including 1 case each in the ARS and NRS groups. Three patients in the ARS group underwent tracheotomy because they could not get off the ventilator postoperatively. Five patients (12.2%) underwent beside continuous renal replacement therapy (CRRT) for postoperative renal failure, including 3 patients in the ARS group and 2 patients in the NRS group. There were 3 cases (7.3%) with poor incision healing, including 1 case in the ARS group and 2 cases in the NRS group. There were 7 cases (17.1%) with mechanical ventilation time > 72 h postoperatively, including 5 cases in the ARS group and 2 cases in the NRS group. The average postoperative intensive care unit (ICU) stay time in the ARS group was (9.5 ± 11.7) days, and the total postoperative hospital stay was (26.4 ± 26.7) days; the NRS group postoperative average ICU stay time was (9.1 ± 12.8) days, and the total postoperative hospital stay was (26.2 ± 26.3) d. There was no statistical difference in postoperative complications between the two groups (Table 3).

**Table 3:** Perioperative results of 41 patients[cases(%)/^−^x ± s]

Variables	Full sample(*n* = 41)	ARS(*n* = 31)	NRS(*n* = 10)	*P* value
Mortality during hospitalization	6 (14.6)	4 (12.9)	2 (20)	0.581
Heart failure	3 (7.3)	1 (3.2)	2 (20)	
Septic shock	3 (7.3)	3 (9.7)	0 (0)	
Cardiopulmonary bypass	168.1 ± 41.2	169.3 ± 42.1	168.0 ± 41.1	0.613
Aortic block time	84.5 ± 22.3	85.6 ± 22.8	85.4 ± 22.2	0.534
Postoperative complications				0.616
Re-exploratory thoracotomy	4 (9.8)	3 (9.7)	1 (10)	
Stroke	2 (4.9)	1 (3.2)	1 (10)	
Tracheotomy	3 (7.3)	3 (9.7)	0 (0)	
CRRT	5 (12.2)	3 (9.7)	2 (20)	
Poor incision healing	3 (7.3)	1 (3.2)	2 (20)	
Mechanical ventilation time > 72 h	7 (17.1)	5 (16.1)	2 (20)	
ICU stay time	9.2 ± 12.5	9.5 ± 11.7	9.1 ± 12.8	0.914
Total postoperative hospital stay	26.3 ± 26.5	26.4 ± 26.7	26.2 ± 26.3	0.896

Abbreviations: *CRRT* continuous renal replacement therapy; *ICU* Intensive Care Unit

Note: Values are presented as n (%) or mean ± standard deviation

### Follow-up results

**Fig. 3:**
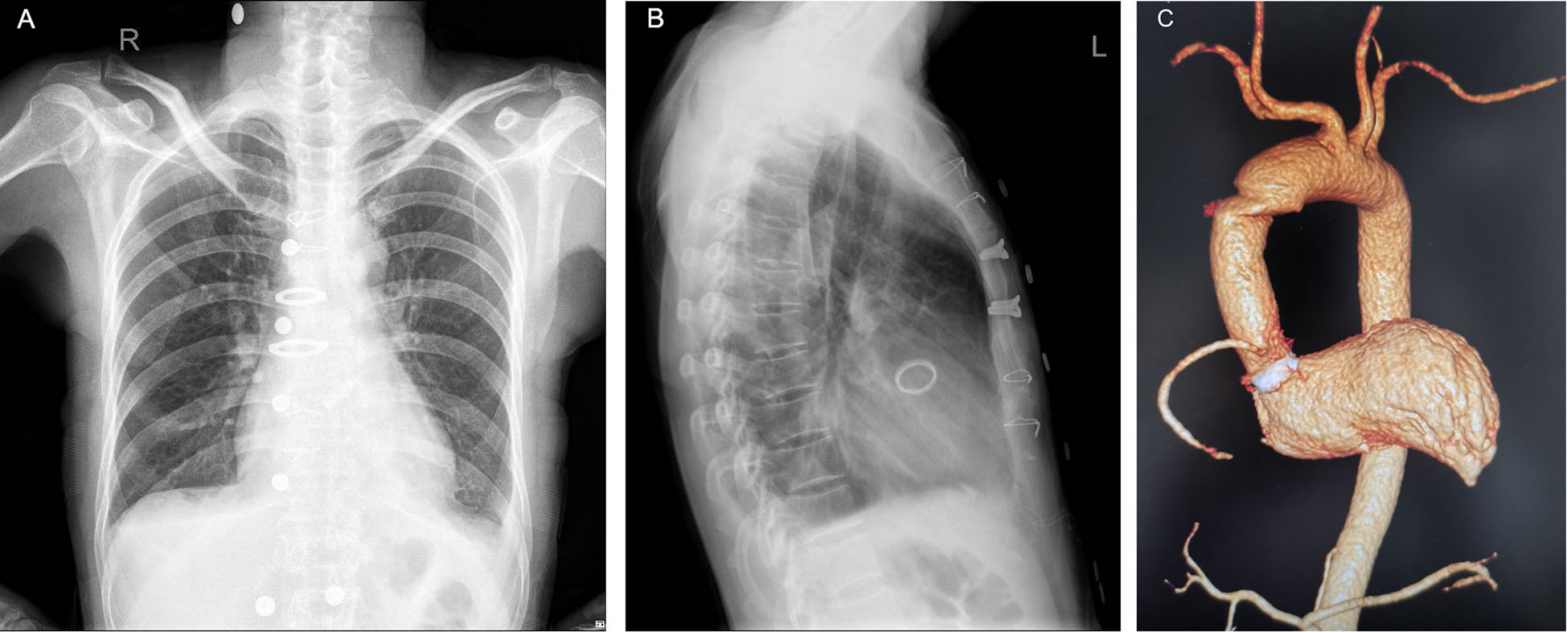
Postoperative anteroposterior (**A**) and lateral (**B**) chest X-rays indicate that the aorta is basically normal after Bentall procedure. Chest CTA (**C**) showed that the artificial vessel and coronary arteries were normal and unobstructed after Bentall procedure

**Fig. 4:**
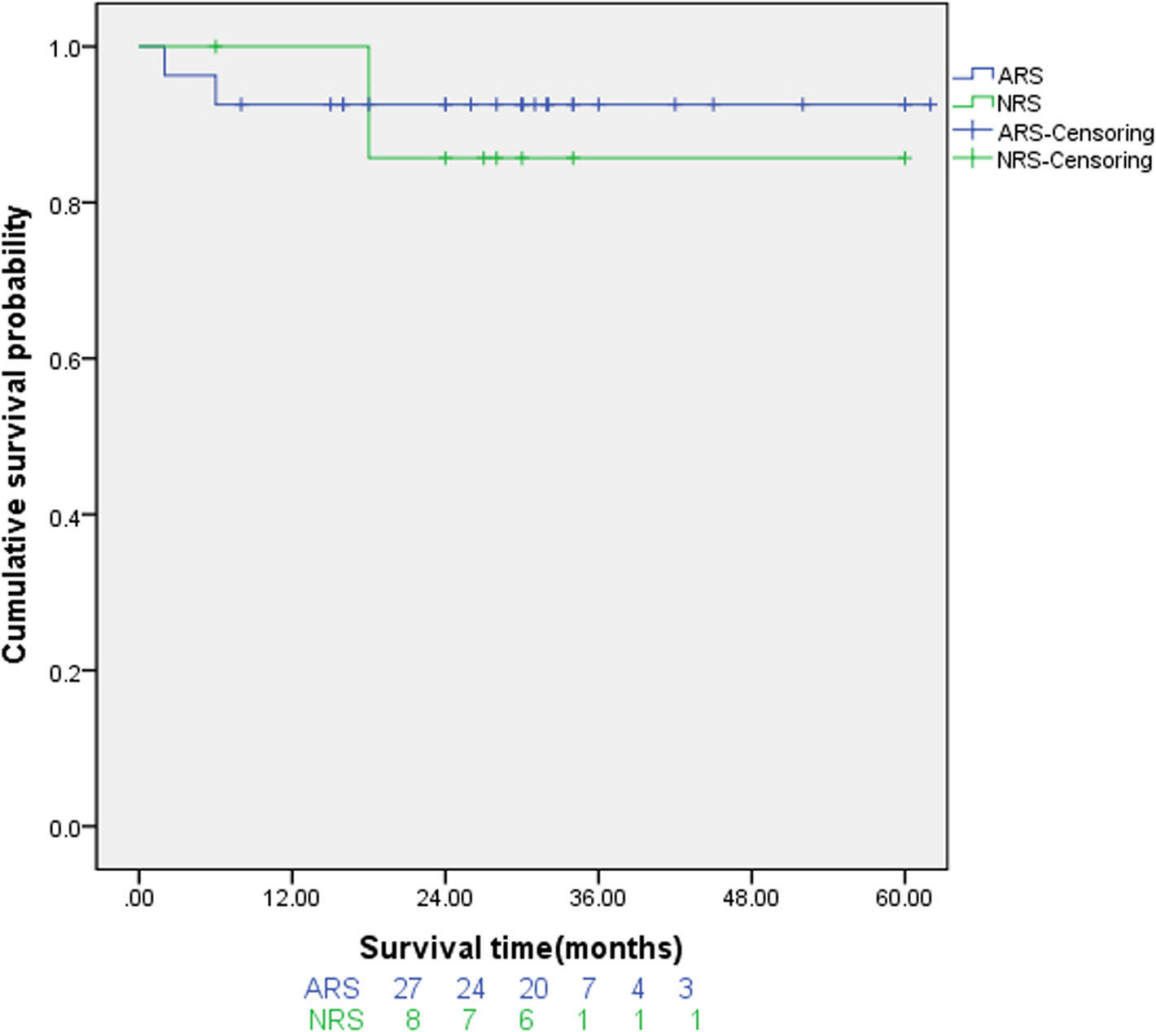
Survival curve of 35 patients underwent redo aortic root replacement after cardiac surgery

The postoperative follow-up period was 3 ~ 62 (30.1 ± 15.5) months. Postoperative chest X-ray and chest CT showed that the artificial blood vessel had a normal shape (Fig. 3). The postoperative heart function (NYHA) of patients improved to grade I or II. The overall 5-year cumulative survival of the two groups was 91.4% (Table 4). In ARS group, 2 patients (7.4%) died during the follow-up period, 1 patient died of septic shock due to mediastinal infection 2 months after discharge, and 1 patient died of cerebral hemorrhage 6 months after discharge. One patient (12.5%) in NRS group died of cerebral hemorrhage 18 months after discharge. The 1-year survival rate (92.6%) of the ARS group was lower than that of the ARS group (100%), while the 3-year survival rate (92.6%) and 5-year survival rate (92.6%) were higher than those of the ARS group (87.5%) (Table 4). However, the overall 5-year cumulative survival rate of the two groups of patients was 91.4%, and there was no statistical difference in overall survival rates between the two groups (*p* = 0.698) (Fig. 4).

**Table 4:** Follow-up results of 35 patients[cases(%)]

Variables	Full sample(*n* = 35)	ARS(*n* = 27)	NRS(*n* = 8)	*P* value
Mortality during follow-up	3 (8.6)	2 (7.4)	1 (12.5)	0.386
Septic shock	1 (2.9)	1 (3.7)	0 (0)	
Cerebral hemorrhage	2 (5.7)	1 (3.7)	1 (12.5)	
1-year cumulative survival		92.6	100	
3-year cumulative survival		92.6	87.5	
5-year cumulative survival	91.4	92.6	87.5	0.698

Note: Values are presented as n (%)

## Discussion

Aortic surgery is becoming more and more mature in our country, and aortic root replacement surgery or Bentall surgery has become a routine surgery in some cardiovascular centers. Some literature reports that the surgical risk of aortic root replacement has been greatly reduced in recent years [2–4]. However, redoing aortic root replacement, especially for patients who have previously unergone aortic root surgery, is still a technically challenging operation [5, 6]. It is reported that the mortality of redo aortic root replacement was as high as 18% [7]. This may be closely related to the patient’s perioperative management, choice of reoperation methods and previous surgical methods. However, our research has found that the hospital mortality of redo aortic root replacement is not necessarily related to the previous operation method. Although the incidence of some serious complications that require reoperation seems to have dropped, the number of patients undergoing reoperation due to various complications has increased, which may be related to the following points. First, with the improvement of imaging technology, some patients with mild or no symptoms have been detected early and received the necessary surgical treatment. Second, the increase in the use of biological valves and the increase in valve-sparing operations have increased the reoperation rate. Third, as the age of patients undergoing cardiac surgery increases, reoperations due to postoperative aortic degeneration also increase.

All cardiac surgeries during thoracotomy are dangerous, especially for patients undergoing reoperation, because the mediastinum and pericardium are fixed due to postoperative adhesions, or the huge ascending aortic aneurysm is close to the sternum, causing the distance between sternum and aorta and right ventricle becomes shorter. If you do not take preventive measures, blindly sawing the sternum, may cause serious consequences such as hemorrhage. It is reported [8] that the complications of re-thoracotomy are still relatively high, although various preventive methods are also clinically applied. Therefore, we recommend that all patients undergoing redo cardiac surgery undergo routine chest X-rays and chest CT examinations before surgery to assess the distance and adhesion between the sternum and the aorta and right ventricle, and take preventive measures based on the examination results. For example, establish peripheral cardiopulmonary bypass firstly before sawing the sternum; routinely place surface defibrillation electrodes before surgery, and perform electrical defibrillation in time when ventricular fibrillation occurs during thoracotomy. Fortunately, in our group of patients, there were no serious complications such as hemorrhage and ventricular fibrillation during re-thoracotomy. Among them, 25 patients (61.0%) established peripheral cardiopulmonary bypass before re-thoracotomy. For patients with a large posterior sternal distance (≥10 mm) or less severe adhesion, we have adopted the strategy of sawing the sternum first and then establishing cardiopulmonary bypass to reduce blood loss during re-thoracotomy. This view is consistent with previous literature report [9].

The main etiologies for this group of patients to redo aortic root replacement after cardiac surgery were true aneurysm, followed by aortic dissection, PVE, PVL and pseudoaneurysm. Further research found that there were 17 cases (54.8%) and 10 cases (100%) in the ARS group and NRS group with true aneurysm of the aortic root/ascending aorta and aortic dissection, respectively. Aortic valve disease combined with dilatation of the ascending aorta is a common clinical problem, because changes in pulsating blood flow, aortic wall disease and Laplace’s law of mechanics increase the risk of aortic rupture [10, 11]. Aortic valve replacement alone can reduce the speed of aortic expansion, but it cannot reduce long-term adverse aortic events after surgery. In patients with preoperative ascending aorta expansion and untreated during the same period, the incidence of aortic adverse events within 10 years was 14.28%, mainly due to the increased risk of aortic dissection and aortic expansion and rupture [12]. At present, there is no gold standard for intervention in ascending aorta diameter expansion, but the overall trend is more positive. The intervention standard from 55 mm recommended by experts to 45 mm recommended by the guidelines indicates that long-term postoperative aortic events are receiving increasing attention [13, 14]. In our group, 20 patients (48.8%) underwent redo cardiac surgery due to dilation of the aortic root and ascending aorta. Among them, 16 patients had preoperative aortic diameter > 55 mm, and the remaining 4 patients had aortic diameter > 50 mm. Unfortunately, we did not collect data on the diameter of the aorta before the previous operation. However, we believe that a more detailed evaluation before the previous operation and simultaneous proper treatment of the dilated ascending aorta should avoid reoperation of some similar patients. Studies [15, 16] reported that aortic disease secondary to aortic valve replacement, especially aortic dissection, has a mortality as high as 44%. The recurrent aortic Stanford type A dissection is mainly broken at the junction of the ascending aorta and the sinus duct. Therefore, Bentall procedure can completely eliminate the breach and reduce the risk of proximal rupture. In this group, 4 patients had ascending aortic dissection after previous aortic valve replacement, and the intimal tear was located at the sinus duct junction in 1 case and the ascending aorta in 3 cases. One case of Debakey II aortic dissection recurrent after aortic dissection surgery, and the intimal tear was in the ascending aorta. The occurrence of these aortic dissections may have a certain relationship with the previous operation. For such patients, before the previous operation, after fully assessing the condition, a more appropriate surgical plan can be selected to avoid the occurrence of reoperation.

In the ARS group, the causes of redo aortic root replacement included 5 cases (12.2%) of PVE, 5 cases (12.2%) of PVL and 4 cases (9.8%) of aortic root pseudoaneurysms. PVE and PVL are important reasons for reoperation after valve replacement, as well as one of the important factors of death during hospitalization. In our study, 10 patients (24.4%) underwent redo aortic root replacement due to PVE and PVL. The main etiologies for PVE and PVL to redo aortic root replacement are as follows: aortic root abscess (3 cases), severe calcification of the aortic sinus wall (2 cases), Behçet disease (4 cases), and aortic sinus wall injury (1 case). Some of these causes are caused by human factors. We can avoid them by improving surgical skills and standardizing surgical operations. Other causes are the patient’s own factors. Regular follow-up can be used to achieve early detection and early treatment. It is reported that in the long-term follow-up after Bentall procedure, the incidence of aortic root pseudoaneurysms was 8 to 15% [17]. In this study, 4 patients (9.8%) with aortic root pseudoaneurysm had Bentall procedure, of which 2 had coronary anastomotic tears, and 2 had valved catheter prosthesis and aortic root avulsion. In the previous operation, it is necessary to fully free the coronary artery opening to prepare button-shaped vascular sheets to ensure that the anastomotic stoma is tension-free to avoid the occurrence of coronary anastomotic stoma tears.

In this group of 41 patients, the hospital mortality was 14.6%. Among them, Among them, the hospital mortality in ARS group was 12.9%, which was lower than the 20% hospital mortality in NRS group. Further research found that the causes of death in ARS group and NRS group were heart failure (3.2% v 20.0%) and septic shock (9.7% v 0%), but there was no statistical difference between the two groups (*p* = 0.581). Previously reported that the mortality of redo aortic root replacement may be related to the previous cardiac surgery method [5–7], but our group of patients was grouped according to the previous cardiac surgery method and found that the mortality of redo aortic root replacement is not necessarily related to the previous cardiac surgery method (*p* > 0.05). We think this may be related to factors such as the severity of the patient’s own disease and the level of the surgeon team. In the ARS group, the main causes of death were septic shock (9.7%) and heart failure (3.2%), while the main cause of death in the NRS group was heart failure (20%). However, there was no statistical difference between the two groups (*p* = 0.581).

Mediastinal infection is one of the main causes of death from redo cardiac surgery, with a hospital mortality as high as 20–50% [18, 19]. In recent years, with the application of negative pressure sealing drainage technology, the treatment effect of mediastinal infection after cardiac surgery has been significantly improved, and the hospital mortality and infection recurrence rate have decreased significantly, but the hospital mortality is still about 5%, and the infection recurrence rate has dropped to 10% or less [20, 21]. Among the 3 patients with septic shock, 2 had mediastinal infection and 1 had lung infection. Among the 2 patients with mediastinal infection, 1 had recurrence of aortic sinus dissection after a previous aortic dissection, which was difficult to stop bleeding, took a long time, had an asthma attack after the tracheal intubation was removed, and had secondary debridement due to mediastinal infection, bedside CRRT was performed due to renal failure, and eventually died of septic shock; another case with Behcet’s disease suffered from paravalvular leakage and received surgical treatment. After the operation, he was given high-dose hormone shock therapy and developed mediastinal infection. Another patient with lung infection was admitted to hospital with acute left heart failure and was assisted by tracheal intubation ventilator. After the operation, the tracheal intubation was removed and the trachea was reintubated due to difficulty breathing, followed by tracheotomy, and finally died of septic shock.

Heart failure is another early cause of death in redo aortic root surgery [3, 4], so appropriate myocardial protection strategies and intraoperative coronary perfusion are particularly important. Among the 3 patients who died of heart failure in this group, 2 patients with aortic dissection involved the right coronary artery opening and had coronary atherosclerotic heart disease. It may be that the insufficient myocardial protection due to intraoperative coronary artery insufficiency, which caused heart failure and eventually lead to death. In redo aortic root replacement, the replantation of the coronary arteries is a key technical issue, especially when the valved tube has been used to replace the aortic root, the coronary anastomosis must be carefully dissected to implant the new graft. At the same time, the anastomosis between the transplanted valved tube and the coronary artery must be tension-free, and the coronary artery must be torsion-free to avoid bleeding or late rupture and pseudoaneurysm. The other one patient was PVE, who underwent emergency surgery due to failure of conservative treatment and eventually died of heart failure.

Therefore, perioperative control of infection and strengthening of nutritional support are one of the important measures to reduce hospital mortality. Therefore, preoperative maintenance of cardiac function and lung function, intraoperative myocardial protection, coronary perfusion, and postoperative infection control measures are conducive to reducing the mortality of redo cardiac surgery.

Most surgeons tend to use mechanical valves for aortic root replacement to avoid reoperation, but the risk of reoperation must be weighed against the risk of stroke and bleeding. Studies have reported that 57% of patients undergo bioprosthetic valve replacement, and this part of patients will be more free from thromboembolic complications after 10 years [22, 23]. Among the 35 follow-up patients in this group, 3 patients (8.6%) died during follow-up, and 2 patients (5.7%) died of cerebral hemorrhage. There was no statistical difference between the ARS group and the NRS group (*p* = 0.386), which may be closely related to improper oral anticoagulant. In elderly patients, a new generation of biological valves may be safer, because biological valve degeneration at this age are relatively rare [24].

This article has the following limitations: (1) The collected patient demographic data lacks information on the patient’s region (rural, urban), education level, income level, etc. These characteristics may be closely related to whether the patient actively participates in the follow-up. (2) This article is a retrospective study has a small sample size and some variables are missing, which may bias the results. Further prospective multi-center large sample studies are needed.

## Conclusions

In summary, redo aortic root replacement is difficult and high risk. For every patient, a sufficient evaluation is required before reoperation, including a suitable surgical approach, adequate myocardial protection, and a complete surgical plan are essential to ensure the success of the reoperation. Bentall procedure is still a reliable surgical option for redo aortic root replacement, with good short- and mid-term results. The method of the previous operation and the etiology of the reoperation are not necessarily related to the prognosis of the patient.

## Data Availability

All datas used during this study can be shared.

## Abbreviations

PVE: Prosthetic valve endocarditis
ECG: Electrocardiogram
CTA: Computed tomography angiology
CPB: Cardiopulmonary bypass
ARS: Aortic root surgery-involved
NRS: Non-aortic root surgery-involved
PVL: Paravalvular leakage
CRRT: Continuous renal replacement therapy
ICU: Intensive care unit
